# The Utility of Zebrafish as a Model for Screening Developmental Neurotoxicity

**DOI:** 10.3389/fnins.2018.00976

**Published:** 2018-12-18

**Authors:** Marta d’Amora, Silvia Giordani

**Affiliations:** ^1^Nano Carbon Materials, Center for Sustainable Future Technologies, Istituto Italiano di Tecnologia, Turin, Italy; ^2^School of Chemical Sciences, Dublin City University, Dublin, Ireland

**Keywords:** zebrafish, model, neurotoxicity, development, chemicals

## Abstract

The developing central nervous system and the blood brain barrier are especially vulnerable and sensitive to different chemicals, including environmental contaminants and drugs. Developmental exposure to these compounds has been involved in several neurological disorders, such as autism spectrum disorders as well as Alzheimer’s and Parkinson’s diseases. Zebrafish (*Danio Rerio*) have emerged as powerful toxicological model systems that can speed up chemical hazard assessment and can be used to extrapolate neurotoxic effects that chemicals have on humans. Zebrafish embryos and larvae are convenient for high-throughput screening of chemicals, due to their small size, low-cost, easy husbandry, and transparency. Additionally, zebrafish are homologous to other higher order vertebrates in terms of molecular signaling processes, genetic compositions, and tissue/organ structures as well as neurodevelopment. This mini review underlines the potential of the zebrafish as complementary models for developmental neurotoxicity screening of chemicals and describes the different endpoints utilized for such screening with some studies illustrating their use.

## Introduction

**FIGURE 1:**
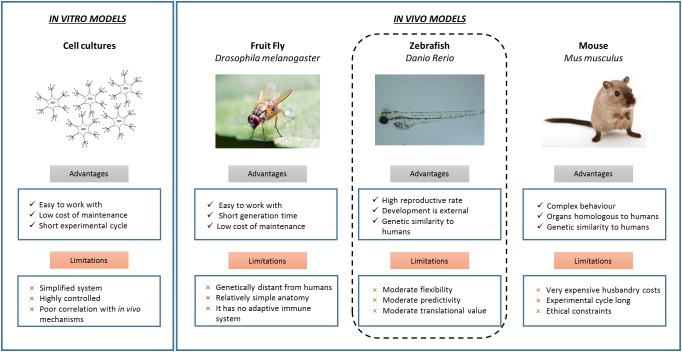
*In vitro* and *in vivo* models for developmental neurotoxicity screening. The possible neurotoxic effects induced by different chemicals can be addressed by using several *in vitro* and *in vivo* systems. *In vitro* cytotoxicity models are cheap and fast, but over-simplified and provide limited and preliminary neurotoxicity data. These findings are implemented performing neurotoxicity tests on *in vivo* animal models. Each *in vivo* system possesses different advantages and limitations. In this framework, zebrafish represent excellent comparative vertebrate systems.

Exposure to different chemicals during development induces a significant risk to human health and can cause the onset of different neurological and neuropsychiatric impairments, ranging from attention deficit hyperactivity disorder (Braun et al., 2006; Bellinger, 2013) to autism spectrum disorders (Harrington et al., 2014; Lyall et al., 2017), and Parkinson’s disease (Barlow et al., 2007). Moreover, various studies indicate that the brain of early-life organisms is more sensitive to chemicals during a critical period in development, including prenatal and postnatal stages (Giussani, 2011; Perera and Herbstman, 2011). The little progress in acknowledging different chemicals to induce neurotoxic effects, is in part due to the disadvantages and restrictions of the different *in vitro* and *in vivo* systems employed to identify adverse effects of chemicals. Different studies have reported the effects induced by different chemicals on the neuronal activity of different cell lines, including mouse and rat primary neuronal cells (Chen et al., 2017; Sethi et al., 2017) and neural precursor cells derived from human induced pluripotent stem cells (Druwe et al., 2015; Ryan et al., 2016). As shown in Figure 1, *in vitro* toxicity studies are cheap, quick and easy, but cultured cells poorly correlate with *in vivo* mechanisms and therefore the observations have limited translational value. Preliminary *in vitro* tests confirm zebrafish as promising candidates for intermediate models. The different advantages of zebrafish are illustrated in Figure 1. Zebrafish are significantly more complex than cultured cells and other model systems, such as *Drosophila melanogaster* (Giacomotto and Segalat, 2010). Moreover, toxicity experiments performed in zebrafish are less expensive and time-consuming than those conducted in rodents (Crofton et al., 2012). Here, we present a brief overview of the different advantages of using zebrafish to assess developmental neurotoxicity and the different endpoints utilized for this screening including some examples illustrating the utiliy of zebrafish studies.

## Zebrafish as Neurotoxicity Models

### Advantages and Limitations

Zebrafish present several advantages for assessing developmental neurotoxicity, making them excellent *in vivo* models in this field (Garcia et al., 2016; Kalueff et al., 2016; Wiley et al., 2017). Zebrafish are small-sized animals and, therefore, can be handled easily. They undergo external fertilization with a high fecundity rate, generating large numbers of embryos. Because of their small size, neurotoxicity tests are generally performed by placing embryos in 96 multi-well plates which reduces the amount of waste and chemicals used, as well as cost. Zebrafish are simply soaked in chemical solutions and the compounds penetrate the transparent embryo’s external membrane by passive diffusion (d’Amora et al., 2016, 2017, 2018). Hence, zebrafish embryos are ideal for high-throughput screening (Horzmann and Freeman, 2018). Another advantage of using zebrafish is that brain development occurs within 3 days post-fertilization, together with the central nervous system. Zebrafish possess a high degree of genetic, morphological and physiological homology with humans (Howe et al., 2013; Kalueff et al., 2014). In particular, development processes and mechanisms of the central nervous system of zebrafish and other vertebrates are well-conserved (Belousov, 2011). The similarity between these species also includes the development of the blood brain barrier (BBB). This is very important, as the BBB plays a crucial role in protecting the brain against chemical substances (Eliceiri et al., 2011). For instance, the counterparts of many brain subdivisions found in the developing mammalian brain are morphologically identifiable in the developing zebrafish (Wullimann, 2009). Thanks to all these features, particularly to the fast brain development, zebrafish are increasingly utilized as complementary models for *in vivo* neurotoxicity screening (Fan et al., 2010; Cowden et al., 2012).

However, there are several peculiarities that may limit their use. The most obvious drawback of zebrafish, specifically in comparison with humans, is that they are not mammals. It is not possible to fully control the chemical dose absorbed since zebrafish embryos are not developing inside a placenta and are exposed to chemicals in medium and absorb them directly (Rubinstein, 2006). Furthermore, chemicals can be metabolized in a different manner compared to mammals. In early life stages, zebrafish are surrounded by a protective membrane which may limit the diffusion of some chemicals (Cudd, 2005). In addition, non-water soluble chemicals cannot be easily dispersed in the embryo medium and thus a small amount of solvent has to be used (Maes et al., 2012).

### Neurotoxicity Endpoints

Considering the positive features of zebrafish described above, several approaches have been developed to utilize zebrafish in neurotoxicity screening during the last decade. Effects of different chemicals on brain development can be evaluated by different neurotoxicity endpoints including gene expression patterns, neural morphogenesis and neurobehavioral profiling (Kalueff et al., 2013; Truong et al., 2014; Chueh et al., 2017).

#### Gene Expression Patterns

A quick and sensitive method to detect changes in gene expression patterns in zebrafish treated with chemicals, is to quantify markers related to developmental toxicity. Fan et al. (2010) used various nervous system genes as potential markers of neurotoxicity, characterzing their expression profiles in embryos exposed to ethanol by means of quantitative real-time polymerase chain reactions. These markers include the transcripts of genes expressed in neuronal stem cells and/or in developing neurons. Their results showed a decrease or increase of these transcripts during development, and in particular highlighted a significant overexpression of a specific astrocytes marker. This study clearly demonstrates that analyzing the brain gene expression profile is a useful tool to rapidly test the neurotoxicity of chemicals during development (Fan et al., 2010). Studies performed in mammals, including mice (Alfonso-Loeches et al., 2013), rats (Gonzalez et al., 2007), and humans (Jung et al., 2010), reported a similar increase in astrocyte marker expression after treatment with ethanol. This approach to assess the chemical profiling expression of a high number of genes, provide knowledge on how different chemicals affect the developing nervous system.

Yang et al. (2007) treated zebrafish embryos with various concentrations of environmental toxins and analyzed the changes in the profiling expression of hundreds of genes by microarray hybridization. The obtained expression profiles were highly specific for each tested compound, allowing to identify several chemicals from the expression profiles with high probability. This study demonstrated that organ and cell-specific changes in gene expression could be detected by *in situ* hybridization (Yang et al., 2007).

Following the work of Yang, the group of Ho et al. (2013) focused on the effects of methyl mercury in the nervous system. A genome profiling analysis of treated zebrafish was carried out in conjunction with whole-mount *in situ* studies of affected genes. An altered expression of various genes involved in different biological functions was found in different neuronal subregions of the brain (Ho et al., 2013).

The gene expression of myelin basic proteins (MBP) was evaluated in zebrafish after treatment with different concentrations of propofol, an anesthetic. The results indicated propofol to be toxic, causing a high decrease in MBP expression levels in the larval central nervous system. In addition, the effects of ibuprofen, diclofenac and paracetamol were assessed by adifferent neuron related expression genes. Ibuprofen and diclofenac exposure down-regulated the *neurog1* expression, while ibuprofen up-regulated it (Xia et al., 2017). Li et al. (2018) assessed the expression of neurodevelopmental genes (*mbp*, *syn2a*, and *α1-tubulin*) in larvae treated with Tris (1,3-dichloro-2-propyl) phosphate and chlorpyrifos (CPF), finding the expression to be down-regulated. The neurotoxicity of triphenyl phosphate was investigated by analyzing the expression of genes which are related to neurodevelopment. Exposure caused down-regulation of *1-tubulin*, *mbp*, *syn2a*, *shha*, and *elavl3*, demonstrating the neurotoxic effects of this organophosphate ester (Shi et al., 2018). Embryos and larvae treated with perfluorododecanoic acid resulted in several down-regulated genes, including *gap43*, *α1-tubulin*, *gfap*, *mbp*, and *elavl3.*

The use of gene profiling patterns represents a useful neurotoxicity endpoint to assess the potential developmental neurotoxicity of different compounds. However, it is important to assure any modifications in gene patterns are caused by the treatment itself and are not a possible stress response (Spurgeon et al., 2010).

#### Neural Morphogenesis

Different research groups have employed zebrafish to address the effect of chemicals on the central nervous system during the development by morphometric endpoints (Scalzo and Levin, 2004; Parng et al., 2006, 2007). Parng et al. (2006) investigated the biological consequences of different compounds, demonstrating their significant neuroprotective effects in zebrafish. They proposed a new *in vivo* approach based on evaluation of oxidation-induced apoptosis. The same group tested the neurotoxicity of different drugs, investigating neuronal apoptosis and other parameters by *in situ* hybridization and immunostaining techniques (Parng et al., 2007). In both studies, obtained data were correlated with previous ones performed in mammals, validating this comparative *in vivo* system for screening. Evaluation of neuronal apoptosis by acridine orange staining was used as an endpoint to evaluate the neurotoxicity of seven compounds. Three of these chemicals caused specific neurotoxicity in catecholaminergic neurons (Ton et al., 2006). In this case, results were comparable with the mammalian studies. In other works, zebrafish eggs were treated with cypermethrin and its toxic effects on the developing nervous system were evaluated (Shi et al., 2011). Notable signs of apoptosis were observed in the nervous system. Acridine orange staining was also employed to explore the neurotoxicity of fenvalerate (Gu et al., 2010), which caused apoptosis in the brain of embryos and larvae and an alteration in neurodevelopmental genes, leading to brain impairment.

In this framework, another approach analyzes axonal morphology and growth during neuronal development (Yang et al., 2011). Zebrafish embryos were treated with the organophosphorus pesticide chlorpyrifos (CPF) or its oxon metabolite (CPFO) and the *in vivo* profiling of axonal growth was evaluated. The results showed an inhibition of the axonal growth in primary motoneurons (PMNs) and secondary motoneurons (SMNs), with consequent anomalies in swimming ability. Muth-Köhne et al. (2012) proposed to determine alterations in zebrafish treated with chemicals as a valid method to investigate their neurotoxic effects. To this end, they developed a novel assay based on whole-mount immunostaining of motorneurons using specific antibodies for PMNs and SMNs. The neurotoxic effects induced by thiocyclam, cartap and disulfiram were analyzed. From the three neurotoxins, disulfiram resulted to be the most toxic and thiocyclam the least.

Another morphometric endpoint, commonly used in developmental toxicity assessment, is the *in vivo* observation of morphological defects in the developing brain. The transparency of zebrafish is one of their peculiarities, helping to observe all brain cells beginning at early stages. Moreover, it is possible to label and visualize *in vivo* specific neurons and subsets of axonal tracts by dye microinjection (d’Amora et al., 2016).

Panzica-Kelly et al. (2010) proposed a morphological score system to distinguish the defects induced by different chemical exposures. They analyzed over 30 chemicals and found changes of morphology or size in one or more brain regions of treated zebrafish.

The potential neurotoxicity of triclosan (TCS) on zebrafish, was evaluated by analyzing morphological changes and expression of genes involved in neurodevelopment. Embryos treated with TCS were affected in their CNS structure, with a decrease in synaptic density and axon length. Moreover, expression of *α1-Tubulin* and *Gap43*, involved in axon extension, were up-regulated, while expression of *Gfap* and *Mbp*, involved in axon myelination, were decreased (Kim et al., 2018).

#### Neurobehavioral Profile

Neurobehavioral changes are the most common neurotoxic endpoints investigated and addressed in zebrafish exposed to chemicals. In particular, the number of movements, spontaneous or induced by stimulation (response to touch), and swimming activity are analyzed. Due to all zebrafish peculiarities, it is easily possible to track *in vivo* behaviors, using video recording tools.

As in mammals, treating zebrafish with ethanol led to altered swimming activity; in particular, ethanol concentrations of 0.5–1% resulted in hyperactivity, while higher doses caused sedation.

Different studies evaluating a possible neurotoxicity of organophosphorus pesticides reported neurobehavioral changes in zebrafish (Eddins et al., 2010; Yen et al., 2011). In particular, larvae exposure to chlorpyrifos, diazinon, and parathion reduced acetylcholine esterase activity and larval motility. Other pesticides also induced various neurobehavior changes (DeMicco et al., 2010; Liu et al., 2018). Zebrafish larvae treated with different pyrethroids presented neurotoxicity characterized by increased motility (DeMicco et al., 2010; Liu et al., 2018). Triphenyl phosphate, an environmental toxicant, was found to significantly reduce larval locomotor activity (Shi et al., 2018). Weichert et al. investigated the consequences of four different chemicals by quantifying spontaneous locomotion. Their results demonstrated the advantages of using behavioral parameters in detecting neurotoxic effects, in particular when exposed to a chemical with a specific mode of action (Weichert et al., 2017).

Xiao et al. (2018) evaluated the effects of 17 typical fluoroquinolones on zebrafish and reported four different types of neurobehaviors with no influence on locomotor activity, suppression of activity or intermediate responses.

Moreover, different approaches were proposed to test locomotor activity by evaluating tail contractions, touch-response, and swimming activity in response to chemicals in the microplate format (Kokel et al., 2010; Selderslaghs et al., 2010). The effects of endosulfan I and endosulfan sulfate were characterized in zebrafish by touch response. Larvae treated with acute doses of both compounds presented a reduced response to touch and in some cases, paralysis (Stanley et al., 2009). Irons et al. (2010) developed another drug challenge paradigm for larvae in a microplate format, using alternating light and dark periods, in order to monitor the neurobehavior much quicker. In the same year, Selderslagh et al. developed new methods to evaluate locomotor activity in zebrafish. Spontaneous tail coilings and swimming of embryos treated with chlorpyrifos, a common pesticide, were evaluated using video recording tools (Selderslaghs et al., 2010). Subsequently, they evaluated this method at several developmental stages, investigating the neurotoxic effects of well-known compounds (Selderslaghs et al., 2013). A classification of these chemicals as being neurotoxic or non-neurotoxic obtained in zebrafish showed a 90% similarity with previous data found in mammals (Selderslaghs et al., 2013).

The behavioral effects of benzo[a]pyren were assessed by means of a larval photomotor response assay. This assay allowed tracking the movements over alternating light and dark periods (Knecht et al., 2017). The highest dose of benzo[a]pyrene (4 μM) caused significant hyperactivity. On the other hand, zebrafish exposed to mercury chloride presented a decrease in the number of tail coilings (Abu Bakar et al., 2017).

### Connections Zebrafish/Humans

Zebrafish assays represent intermediate model systems, that enable high-throughput screening of different chemicals. The use of zebrafish in neurotoxicity research is increasing and different studies underline how these animals can be employed to detect risks for human health, avoiding the ethical constraints of mouse and rat experiments. In this review, we provided multiple examples, from different research groups, using zebrafish as promising models to predict the neurotoxicity of chemicals in mammals, including humans.

However, our understanding of the potential neurotoxicity of chemicals during development has not progressed much. One of the reasons for this is the lack of a common protocol used by researchers; in fact the concentrations of chemicals, the temporal window of chemical exposure, and the method of statistical analyses are different. So far, standard criteria for neurotoxicity are missing.

A systematic comparison of chemical neurotoxicity in zebrafish and mammals is necessary to validate zebrafish as alternative model for human toxicology. Such data will convince chemical companies of the potency and benefits of zebrafish as predictors of neurotoxic effects in humans. We believe that zebrafish will gain more attention and they will become highly popular organisms for testing chemicals.

## Conclusion

This mini review gives a brief overview of the potential use of zebrafish to evaluate neurotoxicity during development. Zebrafish possess significant advantages as model organisms and can overcome the limitations of other systems, making them potentially suitable as models in neurotoxicology. Thanks to their peculiarities, zebrafish can be employed as outstanding platforms to efficiently and rapidly evaluate the impact of chemicals on the developing brain. They offer the possibility to screen several toxicity endpoints by combining different assays, allowing to generate quantitative assessments of a large numbers of chemicals. We believe, the increasing employment of zebrafish in testing chemicals will speed up this process and facilitate the understating of neurotoxicity mechanisms.

## Author Contributions

All authors listed have made a substantial, direct and intellectual contribution to the work, and approved it for publication.

## Conflict of Interest Statement

The authors declare that the research was conducted in the absence of any commercial or financial relationships that could be construed as a potential conflict of interest.
